# Methyl 2,6-bis­[(5-bromo-4,6-dimeth­oxy­pyrimidin-2-yl)­oxy]benzoate

**DOI:** 10.1107/S1600536810024724

**Published:** 2010-06-30

**Authors:** Hoong-Kun Fun, Jia Hao Goh, Sankappa Rai, Arun M. Isloor, Prakash Shetty

**Affiliations:** a X-ray Crystallography Unit, School of Physics, Universiti Sains Malaysia, 11800 USM, Penang, Malaysia; b Department of Chemistry, Manipal Institute of Technology, Manipal University, Manipal 576 104, India; c Organic Chemistry Division, Department of Chemistry, National Institute of Technology-Karnataka, Surathkal, Mangalore 575 025, India; d Department of Printing, Manipal Institute of Technology, Manipal University, Manipal 576 104, India

## Abstract

In the title compound, C_20_H_18_Br_2_N_4_O_8_, the inter­planar angle of the pyrimidine rings is 75.1 (2)°. The central benzene ring is inclined at inter­planar angles of 66.5 (2) and 71.9 (2)° with respect to the two pyrimidine rings. In the crystal structure, adjacent mol­ecules are connected into two-mol­ecule-thick arrays parallel to the *bc* plane *via* short Br⋯Br [3.5328 (12) Å] and Br⋯O [3.206 (3) and 3.301 (4) Å] inter­actions. A weak inter­molecular π–π aromatic stacking inter­action [centroid–centroid distance = 3.526 (3) Å] is also observed.

## Related literature

For general background to and applications of the title compound, see: Koichiro *et al.* (1988 ▶); He *et al.* (2007 ▶); Li *et al.* (2006 ▶); Gerorge (1983 ▶). For closely related structures, see: Fun *et al.* (2010 ▶); Li & Luo (2006 ▶).
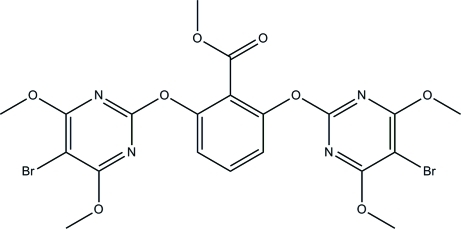


## Experimental

### 

#### Crystal data

C_20_H_18_Br_2_N_4_O_8_
*M* *_r_* = 602.20
Monoclinic, 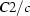

*a* = 29.972 (5) Å
*b* = 8.1392 (12) Å
*c* = 23.061 (3) Å
β = 123.120 (3)°
*V* = 4711.8 (12) Å^3^
*Z* = 8
Mo *K*α radiation
μ = 3.49 mm^−1^
*T* = 293 K
0.20 × 0.18 × 0.14 mm

#### Data collection

Bruker APEXII DUO CCD diffractometer
Absorption correction: multi-scan (*SADABS*; Bruker, 2009 ▶) *T*_min_ = 0.549, *T*_max_ = 0.640
25204 measured reflections
8438 independent reflections
4458 reflections with *I* > 2σ(*I*)
*R*_int_ = 0.066

#### Refinement

*R*[*F* ^2^ > 2σ(*F* ^2^)] = 0.064
*wR*(*F* ^2^) = 0.254
*S* = 1.02
8438 reflections
306 parameters
H-atom parameters constrained
Δρ_max_ = 1.32 e Å^−3^
Δρ_min_ = −1.56 e Å^−3^

### 

Data collection: *APEX2* (Bruker, 2009 ▶); cell refinement: *SAINT* (Bruker, 2009 ▶); data reduction: *SAINT*; program(s) used to solve structure: *SHELXTL* (Sheldrick, 2008 ▶); program(s) used to refine structure: *SHELXTL*; molecular graphics: *SHELXTL*; software used to prepare material for publication: *SHELXTL* and *PLATON* (Spek, 2009 ▶).

## Supplementary Material

Crystal structure: contains datablocks global, I. DOI: 10.1107/S1600536810024724/hb5519sup1.cif

Structure factors: contains datablocks I. DOI: 10.1107/S1600536810024724/hb5519Isup2.hkl

Additional supplementary materials:  crystallographic information; 3D view; checkCIF report

## Figures and Tables

**Fig. 1.**
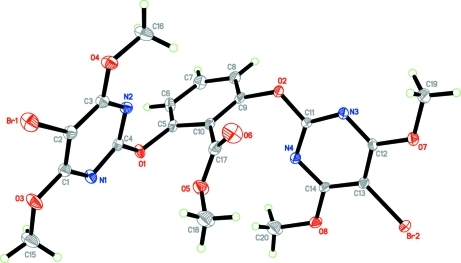
The molecular structure of (I), showing 20 % probability displacement ellipsoids for non-H atoms.

**Fig. 2.**
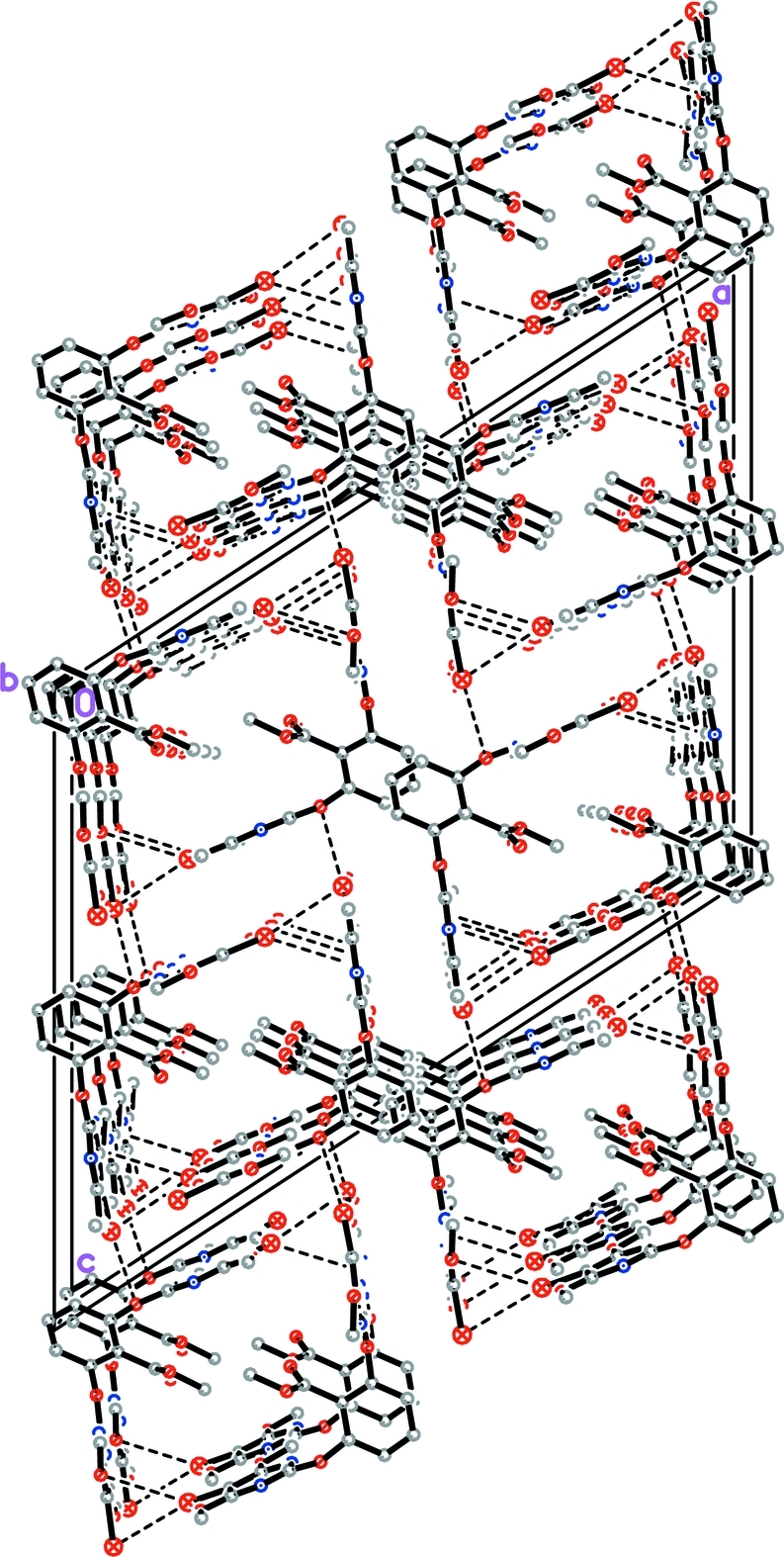
The crystal structure of (I), viewed along the b axis, showing two-molecule-wide arrays parallel to the bc plane. H atoms not involved in intermolecular interactions (dashed lines) have been omitted for clarity.

## supplementary crystallographic information

### Comment

Methyl-2,6-bis[(5-bromo-4,6-dimethoxypyrimidin-2-yl)oxy]benzoate is a derivative of herbicide showing excellent herbicidal effects on annual and perennial weeds and high-safety crops, especially rice and wheat and is applied to paddy fields, ploughed fields and non-agricultural land (Koichiro *et al.*, 1988). Most sulphonylurea herbicides and all pyrimidinylbenzoate herbicides (He *et al.*, 2007) such as nicofulfuron, amidosulfuron, halopyrazosulfuron, ethoxysulfuron, pyriminobac-methyl and pyriftalid, possess 4,6-dimethoxypyrimidin-2-yl groups (Li *et al.*, 2006), while sulfometuron-methyl, a kind of sulfonylurea, contains 4,6-dimethylpyrimidin-2-yl groups, which suggests that the two disubstituted pyrimidin-2-yl groups possess high biological activity (Gerorge, 1983).

In the title compound (Fig. 1), the two pyrimidine rings with atom sequences N1/C1/C2/C3/N2/C4 and C11/N3/C12/C13/C14/N4 are essentially planar, with maximum deviations of -0.028 (6) and 0.010 (5) Å, respectively, at atoms C1 and N4. An interplanar angle of 75.1 (2)° is formed between these two pyrimidine rings. The central phenyl ring (C5-C10) is inclined at interplanar angles of 66.5 (2) and 71.9 (2)°, respectively, with respect to the N1/C1/C2/C3/N2/C4 and C11/N3/C12/C13/C14/N4 pyrimidine rings. The geometric parameters agree well with those observed in closely related structures (Fun *et al.*, 2010; Li & Luo, 2006).

In the crystal structure, no classical hydrogen bond is observed. The interesting features of the crystal structure are the intermolecular short Br···Br [Br1···Br2^i^ = 3.5328 (12) Å; (i) -x+1/2, y-1/2, -z+1/2] and Br···O [Br1···O8^i^ = 3.301 (4) and Br2···O1^ii^ = 3.206 (3) Å; (ii) x, -y+2, z+1/2] interactions, which are shorter than the sum of the Van der Waals radii of the relevant atoms, interconnecting adjacent molecules into two-molecule-thick arrays parallel to the *bc* plane. Weak intermolecular π–π aromatic stacking interactions [*Cg*1···*Cg*1^iii^ = 3.526 (3) Å; (iii): -x, y, -z+1/2] involving the C11/N3/C12/C13/C14/N4 pyrimidine ring further stabilize the crystal structure.

### Experimental

To a stirred solution of methyl-2,6-dihydroxybenzoate (0.50 g, 0.0026 mol) in acetonitrile (10 ml) was added potassium carbonate (1.00 g, 0.0070 mol) and 5-bromo-4,6-dimethoxy-2-(methylsulfonyl)pyrimidine (1.78 g, 0.0050 mol). The reaction mixture was heated to reflux for 4 h. Mass analysis showed completion of the reaction. The reaction mixture was filtered and filtrate was concentrated.  The residue was recrystallized using dichloromethane to obtain brown blocks of (I) (Yield: 67 %, *M.p.* 440–443 K).

### Refinement

All H atoms were placed in their calculated positions, with C—H = 0.93 – 0.96 Å, and refined using a riding model with *U*_iso_ = 1.2 or 1.5 *U*_eq_(C). The rotating group model was used for the methyl groups.

### Crystal data

**Table:**

C_20_H_18_Br_2_N_4_O_8_	*F*(000) = 2400
*M**_r_* = 602.20	*D*_x_ = 1.698 Mg m^−^^3^
Monoclinic, *C*2/*c*	Mo *K*α radiation, λ = 0.71073 Å
Hall symbol: -C 2yc	Cell parameters from 2909 reflections
*a* = 29.972 (5) Å	θ = 2.7–25.5°
*b* = 8.1392 (12) Å	µ = 3.49 mm^−^^1^
*c* = 23.061 (3) Å	*T* = 293 K
β = 123.120 (3)°	Block, brown
*V* = 4711.8 (12) Å^3^	0.20 × 0.18 × 0.14 mm
*Z* = 8	

### Data collection

**Table:**

Bruker APEXII DUO CCD diffractometer	8438 independent reflections
Radiation source: fine-focus sealed tube	4458 reflections with *I* > 2σ(*I*)
graphite	*R*_int_ = 0.066
φ and ω scans	θ_max_ = 32.5°, θ_min_ = 2.7°
Absorption correction: multi-scan (*SADABS*; Bruker, 2009)	*h* = −45→45
*T*_min_ = 0.549, *T*_max_ = 0.640	*k* = −12→12
25204 measured reflections	*l* = −34→34

### Refinement

**Table:**

Refinement on *F*^2^	Primary atom site location: structure-invariant direct methods
Least-squares matrix: full	Secondary atom site location: difference Fourier map
*R*[*F*^2^ > 2σ(*F*^2^)] = 0.064	Hydrogen site location: inferred from neighbouring sites
*wR*(*F*^2^) = 0.254	H-atom parameters constrained
*S* = 1.02	*w* = 1/[σ^2^(*F*_o_^2^) + (0.1449*P*)^2^] where *P* = (*F*_o_^2^ + 2*F*_c_^2^)/3
8438 reflections	(Δ/σ)_max_ = 0.001
306 parameters	Δρ_max_ = 1.32 e Å^−^^3^
0 restraints	Δρ_min_ = −1.55 e Å^−^^3^

### Special details

**Table:**

Geometry. All esds (except the esd in the dihedral angle between two l.s. planes) are estimated using the full covariance matrix. The cell esds are taken into account individually in the estimation of esds in distances, angles and torsion angles; correlations between esds in cell parameters are only used when they are defined by crystal symmetry. An approximate (isotropic) treatment of cell esds is used for estimating esds involving l.s. planes.
Refinement. Refinement of F^2^ against ALL reflections. The weighted R-factor wR and goodness of fit S are based on F^2^, conventional R-factors R are based on F, with F set to zero for negative F^2^. The threshold expression of F^2^ > 2sigma(F^2^) is used only for calculating R-factors(gt) etc. and is not relevant to the choice of reflections for refinement. R-factors based on F^2^ are statistically about twice as large as those based on F, and R- factors based on ALL data will be even larger.

### Fractional atomic coordinates and isotropic or equivalent isotropic displacement parameters (Å^2^)

**Table:**

	*x*	*y*	*z*	*U*_iso_*/*U*_eq_	
Br1	0.30639 (2)	0.57188 (7)	0.09822 (4)	0.0594 (2)	
Br2	0.08330 (2)	0.91910 (6)	0.40182 (3)	0.04252 (17)	
O1	0.11548 (12)	0.9112 (3)	0.04513 (18)	0.0337 (7)	
O2	0.05069 (15)	0.5753 (3)	0.15875 (19)	0.0372 (7)	
O3	0.28301 (15)	0.9322 (4)	0.0904 (3)	0.0563 (11)	
O4	0.20456 (14)	0.4280 (4)	0.0769 (2)	0.0459 (9)	
O5	0.16295 (15)	0.8884 (5)	0.1855 (2)	0.0568 (10)	
O6	0.16019 (17)	0.6258 (6)	0.2086 (3)	0.0729 (14)	
O7	0.07542 (16)	0.5580 (4)	0.3707 (2)	0.0422 (8)	
O8	0.06803 (15)	1.0608 (4)	0.27029 (19)	0.0409 (8)	
N1	0.19889 (16)	0.9262 (4)	0.0691 (2)	0.0377 (9)	
N2	0.15798 (13)	0.6659 (4)	0.0595 (2)	0.0324 (8)	
N3	0.06285 (16)	0.5635 (4)	0.2625 (2)	0.0334 (8)	
N4	0.05992 (14)	0.8206 (4)	0.21251 (19)	0.0320 (7)	
C1	0.24118 (18)	0.8474 (6)	0.0787 (3)	0.0378 (10)	
C2	0.24567 (18)	0.6781 (6)	0.0836 (3)	0.0389 (10)	
C3	0.20209 (17)	0.5919 (5)	0.0728 (3)	0.0352 (9)	
C4	0.15971 (16)	0.8269 (5)	0.0587 (2)	0.0311 (9)	
C5	0.07637 (16)	0.8238 (5)	0.0466 (2)	0.0314 (9)	
C6	0.02573 (18)	0.8241 (6)	−0.0133 (3)	0.0410 (11)	
H6A	0.0198	0.8777	−0.0526	0.049*	
C7	−0.01538 (19)	0.7465 (6)	−0.0153 (3)	0.0472 (12)	
H7A	−0.0494	0.7494	−0.0554	0.057*	
C8	−0.00662 (19)	0.6642 (6)	0.0420 (3)	0.0410 (11)	
H8A	−0.0343	0.6082	0.0405	0.049*	
C9	0.04382 (18)	0.6656 (5)	0.1021 (2)	0.0337 (9)	
C10	0.08581 (17)	0.7468 (5)	0.1067 (2)	0.0314 (8)	
C11	0.05805 (17)	0.6583 (5)	0.2136 (2)	0.0310 (8)	
C12	0.06957 (17)	0.6433 (5)	0.3175 (2)	0.0315 (8)	
C13	0.07150 (16)	0.8121 (5)	0.3220 (2)	0.0318 (9)	
C14	0.06590 (16)	0.8963 (5)	0.2677 (2)	0.0300 (8)	
C15	0.2797 (3)	1.1092 (7)	0.0878 (5)	0.068 (2)	
H15A	0.3105	1.1541	0.0909	0.101*	
H15B	0.2780	1.1486	0.1259	0.101*	
H15C	0.2482	1.1428	0.0450	0.101*	
C16	0.1604 (2)	0.3438 (6)	0.0691 (4)	0.0586 (17)	
H16A	0.1653	0.2277	0.0675	0.088*	
H16B	0.1285	0.3779	0.0269	0.088*	
H16C	0.1575	0.3686	0.1076	0.088*	
C17	0.1402 (2)	0.7456 (6)	0.1719 (3)	0.0437 (11)	
C18	0.2141 (3)	0.9103 (8)	0.2484 (4)	0.0720 (14)	
H18A	0.2214	1.0256	0.2574	0.108*	
H18B	0.2409	0.8602	0.2438	0.108*	
H18C	0.2141	0.8600	0.2860	0.108*	
C19	0.0734 (3)	0.3785 (6)	0.3658 (4)	0.0590 (16)	
H19A	0.0695	0.3336	0.4013	0.089*	
H19B	0.1057	0.3382	0.3717	0.089*	
H19C	0.0436	0.3459	0.3212	0.089*	
C20	0.0691 (3)	1.1479 (9)	0.2166 (4)	0.0720 (14)	
H20A	0.0732	1.2634	0.2267	0.108*	
H20B	0.0364	1.1289	0.1729	0.108*	
H20C	0.0985	1.1093	0.2145	0.108*	

### Atomic displacement parameters (Å^2^)

**Table:**

	*U*^11^	*U*^22^	*U*^33^	*U*^12^	*U*^13^	*U*^23^
Br1	0.0381 (3)	0.0527 (3)	0.0907 (5)	0.0122 (2)	0.0374 (3)	0.0068 (3)
Br2	0.0570 (3)	0.0430 (3)	0.0376 (3)	−0.0108 (2)	0.0324 (2)	−0.0123 (2)
O1	0.0330 (14)	0.0321 (15)	0.0452 (19)	0.0082 (11)	0.0272 (14)	0.0116 (13)
O2	0.058 (2)	0.0298 (15)	0.0372 (18)	−0.0026 (13)	0.0347 (17)	−0.0028 (13)
O3	0.0390 (18)	0.0438 (19)	0.096 (3)	−0.0040 (14)	0.043 (2)	−0.0001 (19)
O4	0.0391 (17)	0.0321 (16)	0.068 (3)	0.0026 (12)	0.0305 (18)	−0.0035 (16)
O5	0.048 (2)	0.067 (2)	0.040 (2)	−0.0182 (17)	0.0144 (18)	0.0008 (19)
O6	0.057 (2)	0.075 (3)	0.057 (3)	0.013 (2)	0.012 (2)	0.024 (2)
O7	0.062 (2)	0.0343 (17)	0.040 (2)	−0.0001 (14)	0.0346 (18)	0.0038 (14)
O8	0.056 (2)	0.0282 (15)	0.038 (2)	0.0019 (13)	0.0257 (17)	−0.0022 (14)
N1	0.0375 (19)	0.0338 (19)	0.048 (2)	0.0027 (14)	0.0270 (19)	0.0063 (17)
N2	0.0304 (16)	0.0305 (17)	0.036 (2)	0.0020 (13)	0.0180 (15)	−0.0021 (15)
N3	0.0444 (19)	0.0308 (18)	0.035 (2)	−0.0017 (14)	0.0279 (18)	−0.0015 (15)
N4	0.0391 (18)	0.0312 (17)	0.0285 (19)	0.0000 (14)	0.0202 (16)	0.0004 (15)
C1	0.035 (2)	0.038 (2)	0.042 (3)	0.0017 (17)	0.022 (2)	0.000 (2)
C2	0.035 (2)	0.037 (2)	0.046 (3)	0.0058 (17)	0.023 (2)	−0.001 (2)
C3	0.034 (2)	0.031 (2)	0.040 (3)	0.0046 (15)	0.0196 (19)	0.0021 (18)
C4	0.0302 (18)	0.037 (2)	0.028 (2)	0.0081 (15)	0.0168 (17)	0.0035 (18)
C5	0.0341 (19)	0.035 (2)	0.032 (2)	0.0063 (16)	0.0225 (18)	0.0031 (18)
C6	0.037 (2)	0.052 (3)	0.037 (3)	0.0065 (19)	0.022 (2)	0.011 (2)
C7	0.033 (2)	0.060 (3)	0.040 (3)	0.002 (2)	0.015 (2)	0.000 (2)
C8	0.038 (2)	0.049 (3)	0.038 (3)	−0.0044 (19)	0.022 (2)	−0.006 (2)
C9	0.044 (2)	0.031 (2)	0.036 (2)	0.0029 (16)	0.028 (2)	−0.0070 (18)
C10	0.038 (2)	0.031 (2)	0.029 (2)	0.0024 (16)	0.0204 (18)	−0.0012 (17)
C11	0.039 (2)	0.0274 (19)	0.033 (2)	−0.0021 (15)	0.0233 (19)	−0.0040 (17)
C12	0.0352 (19)	0.035 (2)	0.030 (2)	−0.0022 (16)	0.0214 (18)	0.0028 (18)
C13	0.0311 (18)	0.037 (2)	0.032 (2)	−0.0015 (15)	0.0199 (18)	−0.0054 (18)
C14	0.0306 (18)	0.0305 (19)	0.030 (2)	−0.0004 (14)	0.0169 (17)	−0.0062 (17)
C15	0.065 (4)	0.039 (3)	0.109 (6)	−0.011 (2)	0.055 (4)	−0.006 (3)
C16	0.045 (3)	0.037 (3)	0.093 (5)	−0.001 (2)	0.037 (3)	−0.002 (3)
C17	0.042 (2)	0.055 (3)	0.033 (3)	0.000 (2)	0.020 (2)	−0.004 (2)
C18	0.073 (3)	0.073 (3)	0.053 (3)	−0.018 (2)	0.023 (2)	0.003 (2)
C19	0.104 (5)	0.032 (2)	0.054 (4)	0.000 (3)	0.052 (4)	0.006 (2)
C20	0.073 (3)	0.073 (3)	0.053 (3)	−0.018 (2)	0.023 (2)	0.003 (2)

### Geometric parameters (Å, °)

**Table:**

Br1—C2	1.872 (4)	C5—C6	1.386 (7)
Br2—C13	1.887 (4)	C5—C10	1.401 (6)
O1—C4	1.368 (5)	C6—C7	1.363 (7)
O1—C5	1.388 (5)	C6—H6A	0.9300
O2—C11	1.342 (5)	C7—C8	1.373 (8)
O2—C9	1.412 (6)	C7—H7A	0.9300
O3—C1	1.324 (6)	C8—C9	1.384 (7)
O3—C15	1.443 (6)	C8—H8A	0.9300
O4—C3	1.337 (5)	C9—C10	1.374 (6)
O4—C16	1.411 (6)	C10—C17	1.496 (7)
O5—C17	1.297 (6)	C12—C13	1.376 (6)
O5—C18	1.433 (8)	C13—C14	1.356 (6)
O6—C17	1.213 (7)	C15—H15A	0.9600
O7—C12	1.336 (5)	C15—H15B	0.9600
O7—C19	1.464 (6)	C15—H15C	0.9600
O8—C14	1.341 (5)	C16—H16A	0.9600
O8—C20	1.442 (8)	C16—H16B	0.9600
N1—C1	1.327 (6)	C16—H16C	0.9600
N1—C4	1.335 (6)	C18—H18A	0.9600
N2—C4	1.312 (6)	C18—H18B	0.9600
N2—C3	1.328 (5)	C18—H18C	0.9600
N3—C11	1.308 (6)	C19—H19A	0.9600
N3—C12	1.339 (6)	C19—H19B	0.9600
N4—C11	1.323 (5)	C19—H19C	0.9600
N4—C14	1.334 (6)	C20—H20A	0.9600
C1—C2	1.383 (6)	C20—H20B	0.9600
C2—C3	1.380 (6)	C20—H20C	0.9600
			
C4—O1—C5	117.6 (3)	N4—C11—O2	118.2 (4)
C11—O2—C9	118.4 (3)	O7—C12—N3	119.6 (4)
C1—O3—C15	118.4 (4)	O7—C12—C13	118.1 (4)
C3—O4—C16	117.6 (4)	N3—C12—C13	122.3 (4)
C17—O5—C18	119.2 (5)	C14—C13—C12	117.1 (4)
C12—O7—C19	118.0 (4)	C14—C13—Br2	122.1 (3)
C14—O8—C20	118.4 (5)	C12—C13—Br2	120.8 (4)
C1—N1—C4	113.8 (4)	N4—C14—O8	118.8 (4)
C4—N2—C3	114.4 (4)	N4—C14—C13	122.2 (4)
C11—N3—C12	114.7 (4)	O8—C14—C13	119.0 (4)
C11—N4—C14	115.3 (4)	O3—C15—H15A	109.5
O3—C1—N1	119.6 (4)	O3—C15—H15B	109.5
O3—C1—C2	117.7 (4)	H15A—C15—H15B	109.5
N1—C1—C2	122.4 (4)	O3—C15—H15C	109.5
C3—C2—C1	116.9 (4)	H15A—C15—H15C	109.5
C3—C2—Br1	121.9 (3)	H15B—C15—H15C	109.5
C1—C2—Br1	121.1 (3)	O4—C16—H16A	109.5
N2—C3—O4	118.6 (4)	O4—C16—H16B	109.5
N2—C3—C2	122.4 (4)	H16A—C16—H16B	109.5
O4—C3—C2	119.0 (4)	O4—C16—H16C	109.5
N2—C4—N1	129.8 (4)	H16A—C16—H16C	109.5
N2—C4—O1	117.6 (4)	H16B—C16—H16C	109.5
N1—C4—O1	112.6 (4)	O6—C17—O5	123.8 (5)
C6—C5—O1	117.0 (4)	O6—C17—C10	124.0 (5)
C6—C5—C10	120.5 (4)	O5—C17—C10	112.2 (4)
O1—C5—C10	122.5 (4)	O5—C18—H18A	109.5
C7—C6—C5	120.6 (5)	O5—C18—H18B	109.5
C7—C6—H6A	119.7	H18A—C18—H18B	109.5
C5—C6—H6A	119.7	O5—C18—H18C	109.5
C6—C7—C8	120.0 (5)	H18A—C18—H18C	109.5
C6—C7—H7A	120.0	H18B—C18—H18C	109.5
C8—C7—H7A	120.0	O7—C19—H19A	109.5
C7—C8—C9	119.3 (4)	O7—C19—H19B	109.5
C7—C8—H8A	120.3	H19A—C19—H19B	109.5
C9—C8—H8A	120.3	O7—C19—H19C	109.5
C10—C9—C8	122.3 (4)	H19A—C19—H19C	109.5
C10—C9—O2	121.0 (4)	H19B—C19—H19C	109.5
C8—C9—O2	116.7 (4)	O8—C20—H20A	109.5
C9—C10—C5	117.2 (4)	O8—C20—H20B	109.5
C9—C10—C17	121.5 (4)	H20A—C20—H20B	109.5
C5—C10—C17	121.2 (4)	O8—C20—H20C	109.5
N3—C11—N4	128.3 (4)	H20A—C20—H20C	109.5
N3—C11—O2	113.5 (3)	H20B—C20—H20C	109.5
			
C15—O3—C1—N1	−4.0 (8)	C8—C9—C10—C17	−179.4 (4)
C15—O3—C1—C2	−177.8 (6)	O2—C9—C10—C17	−1.0 (6)
C4—N1—C1—O3	−179.2 (5)	C6—C5—C10—C9	3.4 (6)
C4—N1—C1—C2	−5.6 (7)	O1—C5—C10—C9	179.2 (4)
O3—C1—C2—C3	178.6 (5)	C6—C5—C10—C17	−179.9 (4)
N1—C1—C2—C3	4.9 (8)	O1—C5—C10—C17	−4.1 (6)
O3—C1—C2—Br1	−5.6 (7)	C12—N3—C11—N4	1.2 (7)
N1—C1—C2—Br1	−179.2 (4)	C12—N3—C11—O2	−179.2 (4)
C4—N2—C3—O4	177.9 (5)	C14—N4—C11—N3	−2.2 (7)
C4—N2—C3—C2	−1.0 (7)	C14—N4—C11—O2	178.3 (4)
C16—O4—C3—N2	−2.1 (7)	C9—O2—C11—N3	178.6 (4)
C16—O4—C3—C2	176.8 (5)	C9—O2—C11—N4	−1.8 (6)
C1—C2—C3—N2	−1.4 (8)	C19—O7—C12—N3	−0.9 (7)
Br1—C2—C3—N2	−177.1 (4)	C19—O7—C12—C13	−180.0 (5)
C1—C2—C3—O4	179.8 (5)	C11—N3—C12—O7	−179.3 (4)
Br1—C2—C3—O4	4.0 (7)	C11—N3—C12—C13	−0.3 (6)
C3—N2—C4—N1	0.0 (7)	O7—C12—C13—C14	179.5 (4)
C3—N2—C4—O1	179.8 (4)	N3—C12—C13—C14	0.4 (6)
C1—N1—C4—N2	3.2 (8)	O7—C12—C13—Br2	1.1 (5)
C1—N1—C4—O1	−176.5 (4)	N3—C12—C13—Br2	−178.0 (3)
C5—O1—C4—N2	12.3 (6)	C11—N4—C14—O8	−179.9 (4)
C5—O1—C4—N1	−167.9 (4)	C11—N4—C14—C13	2.2 (6)
C4—O1—C5—C6	−121.7 (5)	C20—O8—C14—N4	−6.1 (6)
C4—O1—C5—C10	62.4 (5)	C20—O8—C14—C13	171.9 (5)
O1—C5—C6—C7	−177.4 (4)	C12—C13—C14—N4	−1.4 (6)
C10—C5—C6—C7	−1.4 (7)	Br2—C13—C14—N4	177.0 (3)
C5—C6—C7—C8	−1.4 (8)	C12—C13—C14—O8	−179.4 (4)
C6—C7—C8—C9	2.1 (8)	Br2—C13—C14—O8	−1.0 (5)
C7—C8—C9—C10	0.1 (7)	C18—O5—C17—O6	−2.0 (9)
C7—C8—C9—O2	−178.4 (4)	C18—O5—C17—C10	176.5 (5)
C11—O2—C9—C10	72.1 (5)	C9—C10—C17—O6	39.9 (7)
C11—O2—C9—C8	−109.4 (5)	C5—C10—C17—O6	−136.6 (6)
C8—C9—C10—C5	−2.8 (6)	C9—C10—C17—O5	−138.5 (5)
O2—C9—C10—C5	175.7 (4)	C5—C10—C17—O5	44.9 (6)
